# Perception of discrimination by the head of the household and household food insecurity in Venezuelan migrants in Peru: Cross-sectional analysis of a population-based survey

**DOI:** 10.1016/j.pmedr.2025.103050

**Published:** 2025-03-26

**Authors:** Darlene Milagros Rocha-Rojas, Paola Fernanda Velásquez-Huamán, Akram Hernández-Vásquez, Diego Azañedo

**Affiliations:** a Universidad Científica del Sur, Lima, Peru; b Centro de Excelencia en Investigaciones Económicas y Sociales en Salud, Vicerrectorado de Investigación, Universidad San Ignacio de Loyola, Lima, Peru

**Keywords:** Perceived discrimination, Food insecurity, Transients and migrants, Venezuela, Peru

## Abstract

**Objectives:**

To evaluate the association between the perception of discrimination by the head of the household and the presence of moderate to severe food insecurity in the households of Venezuelan migrants and refugees residing in Peru.

**Methods:**

Secondary data analysis of the Survey Directed at the Venezuelan Population Resident in Peru (ENPOVE, for its acronym in Spanish) 2022. The analysis included 3491 migrant Venezuelan family heads. The exposure variable was discrimination perceived by the head of the household (yes/no) since arriving in Peru and the outcome was the presence of moderate to severe food insecurity in the household (yes/no) in the last 30 days, measured with the Food Insecurity Experience Scale. Adjusted prevalence ratios were estimated to evaluate the association of interest, adjusted for potential confounding variables.

**Results:**

Of the total participants, 35.6 % had perceived discrimination since arriving in Peru; Likewise, 39 % of households presented moderate to severe food insecurity. The perception of discrimination by the head of the household was associated with a greater probability of presenting moderate to severe food insecurity in the home (adjusted prevalence ratio: 1.29; 95 % confidence interval [CI]: 1.17–1.42).

**Conclusions:**

The perception of discrimination among Venezuelan migrant and refugee household heads residing in the country is associated with a greater probability of moderate to severe food insecurity. Urgent measures are required to mitigate this problem which will potentially affect food and socioeconomic inequality in our country.

## Highlights

• The discrimination-food insecurity link in Venezuelan migrants in Peru is unknown.
• Perceived discrimination raised the likelihood of moderate-severe food insecurity.
• Results support measures like support groups, legal aid, and nutrition programs.

## 1 Introduction

According to the Food and Agriculture Organization of the United Nations, food insecurity is defined as the lack of access to sufficient, safe, and nutritious food to meet daily energy needs and food priorities for a healthy and active life (Food and Agriculture Organization of the United Nations, 2025). Between 2014 and 2021, a large proportion of the world's population experienced food insecurity (30 %), with a particularly high prevalence in Latin America and the Caribbean (40.6 %), followed, albeit to a lesser extent, by Asia (24.6 %), North America and Europe (8 %) (FAO, IFAD, PAHO, UNICEF, WFP, 2023). In 2021, 16.6 million inhabitants in Peru were reported to have food insecurity, with 6.8 million individuals presenting severe food insecurity (FAO, IFAD, PAHO, UNICEF, WFP, 2023). The pernicious effects of this situation are wide-ranging, including shortages in micronutrient intake and negative health consequences, such as infant emaciation, stunting, noncommunicable diseases, overweight or obesity (Ghani et al., 2023; Castañeda et al., 2019), psychosocial stress, depression (Leung et al., 2015), changes in eating patterns (Coleman-Jensen et al., 2025), skipping meals, and weight loss (Hernández et al., 2013).

Migrants are among the groups most affected by food insecurity due to the social and economic challenges they face in receiving countries (Oyarte et al., 2023; Islam et al., 2022). An estimated eight million Venezuelans have migrated, with 84 % residing Latin American and Caribbean countries (World Development Report, 2023). In 2023, food insecurity affected 97 % of Venezuelan migrants in Chile, 92 % in Brazil, and 49 % in Colombia, due to a lack of economic resources (RMNA, 2023). In Lima, Peru, a 2021 study found food insecurity in 76.3 % of Venezuelan refugee families, with 85 % reporting anxiety about food access and 80 % reducing daily food portions (Action Against Hunger & European Union, 2023). Likewise, a significant percentage of families opted to suppress a meal during the day (61 %) or at least one member of the family went without food for a whole day due to lack of resources to stock up on food (Action Against Hunger & European Union, 2023). In 2022, the figure for food insecurity in the Venezuelan migrant population in metropolitan Lima increased to 96.7 % (Action Against Hunger, 2022). Another problem faced by immigrants is discrimination of any kind by residents of the receiving country. Indeed, 62 % of the Venezuelan population in Peru has perceived discrimination, mainly in public places and the workplace (IDEHPUCP, 2022). Undocumented migrants or those without legal residency are more vulnerable to discrimination, exploitation, food insecurity, and have limited access to national health promotion programs (Blouin et al., 2021). Repeated discrimination worsens physical and mental health (Straiton et al., 2019), affects employment opportunities and well-being (Mera-Lemp et al., 2019), and is linked to depression, low self-esteem, psychosocial stress, and social isolation (Oyarte et al., 2023; Scholaske et al., 2019).

Some studies have explored the relationship between discrimination and food insecurity in diverse contexts. A study of African-American households in South Carolina, United States (US), identified that every one unit increase in the frequency of racial discrimination that participants experienced was associated with a 5 % increase in the likelihood of having very low food security (Burke et al., 2018). A study of female caregivers in the US identified that households with caregivers born outside the US were twice as likely to have food insecurity at home, compared to households of caregivers born in the US. Likewise, Latino and black caregivers were 2.5 and 1.73 times more likely to have food insecurity at home compared to white caregivers born outside the US. Similarly, 35 % of caregivers in households with food insecurity reported having had experiences of discrimination in various social environments (Chilton et al., 2018). Perceived discrimination contributes to food insecurity through economic barriers, limited job opportunities, and lower wages, leading to a negative impact on mental health, which, in turn, affects eating habits (Blouin et al., 2021). In this sense, to date, no study has evaluated the association between the perception of discrimination and food insecurity among Venezuelan immigrants residing in Peru.

In the context of Venezuelan migration in Peru, the National Institute of Statistics and Informatics (INEI, for its acronym in Spanish) has published the results of the second survey conducted aimed at the Venezuelan population residing in Peru (ENPOVE 2022). The objective of ENPOVE was to provide information about the conditions in which Venezuelan migrants live. This survey also covered demographic, social, discrimination and health aspects in order to identify the most urgent needs of this population (INEI, 2022a). Currently, there are several descriptive studies on the food security of Venezuelan migrants in Peru based on socioeconomic and demographic characteristics (Action Against Hunger & European Union, 2021; United Nations, 2021; WFP, 2022; Santandreu et al., 2025). However, it has not been assessed whether the perception of discrimination in the Venezuelan population can affect household food insecurity. Given this context, the objective of this study was to evaluate the association between the perception of discrimination by the head of the household and the presence of moderate to severe food insecurity in Venezuelan migrant and refugee households residing in Peru, using the ENPOVE 2022 database. The evidence generated could provide a framework for the design of national multidisciplinary strategies to mitigate these problems.

## 2 Materials and methods

### 2.1 Ethical considerations

The study did not require the approval of an ethics committee because it involved the analysis of secondary data that are in the public domain and do not allow the identification of the participants evaluated. ENPOVE 2022 was developed by the INEI, and all participants were required to provide verbal informed consent. Data were obtained in an anonymized form from the INEI microdata page: https://proyectos.inei.gob.pe/microdatos/. The present study was approved by the Universidad Científica del Sur with Directorial Resolution No. 012-DGIDI-Cientifica-2021. This project was carried out as part of the requirements to obtain the degree of Medical Doctor.

### 2.2 Study design and population

Observational, analytical cross-sectional study performed using data from the ENPOVE 2022 conducted in Peru by the INEI (INEI, 2022a). The ENPOVE 2022 is a survey performed with the purpose of determining the characteristics of the population of Venezuelan refugees and migrants residing in Peru. The reporting of this manuscript follows the guidelines of the Strengthening the Reporting of Observational studies in Epidemiology declaration (Elm et al. 2007).

Data collection was carried out by personnel hired by INEI. The pollsters were trained to conduct the interviews, which included a series of direct questions to the respondents. Mobile devices (tablets) were used for information collection. In case of not being able to carry out the interviews by phone, they were carried out in person (INEI, 2022b). The ENPOVE 2022 was performed in urban areas of department capitals, where the majority of households with Venezuelan residents are concentrated (82.9 %). The cities chosen were: Metropolitan Lima and Callao, Chimbote, Arequipa, Trujillo, Chiclayo, Tumbes, Ica and Piura (INEI, 2022b).

**Fig. 1:**
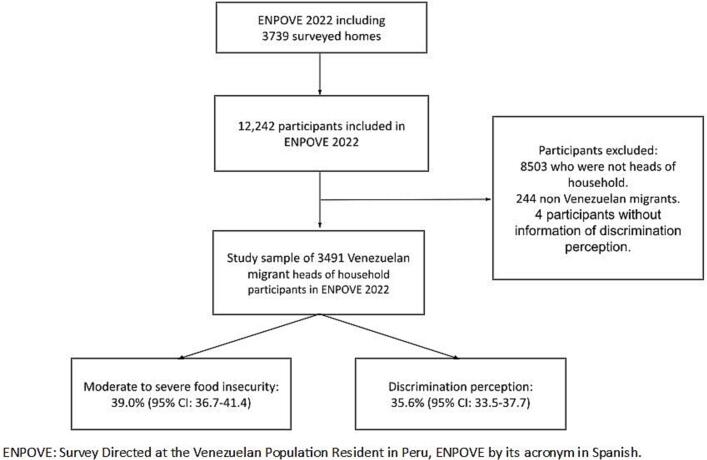
Flow chart of participants included in the study.

ENPOVE 2022 included 3739 Venezuelan households, comprising a total of 12,242 persons (INEI, 2022a). For the present analysis, 3491 household heads were included. In addition, 244 participants who were not Venezuelan migrants and four participants, in whom data on the perception of discrimination were lacking, were excluded (See Fig. 1). Further details on the design, procedures, data collection and questionnaires can be found in the ENPOVE 2022 data sheet (INEI, 2022b).

### 2.3 Variables and measurements

#### 2.3.1 Outcome variable

The outcome variable of our study was “Moderate to severe food insecurity” (Yes/No). This variable was defined based on the questions of the Food Insecurity Experience Scale (FIES), used in the ENPOVE 2022. This questionnaire consists of eight questions related to food insecurity in the household during the last 30 days (See table S1 of the supplementary material).

The items of this scale are dichotomous (yes or no), and were recoded as one for affirmative responses and zero for negative ones (the coding was reversed for item two). For the statistical validation of the scale, we used a Rasch model, based on the “RM.weights” package in R (version 4.2.1) (R Core Team, Vienna, Austria) (FAO, 2016). Evaluation of the pertinence of the items was carried out by means of the infit statistic, which evaluates the discriminatory power of the item; items were considered to have an adequate adjustment to the Rasch model when they presented an infit statistic between 0.7 and 1.3. Scale items with infit statistic values greater than 1.3 were considered for elimination. In case of being eliminated, the Rasch model was performed again to reevaluate its validity, following the same procedure. After estimation of the model, it yielded an infit value greater than 1.5 for item two of the scale, and thus, this item was excluded from the analysis. Lastly, the final score was obtained by adding the scores obtained for each answer of the seven questions that showed adequate discriminatory performance (Leung et al., 2015; Hernández-Vásquez et al., 2023). A household was considered to have moderate to severe food insecurity with a score ≥ four points (Hernández-Vásquez et al., 2023; Jankhotkaew et al., 2022).

#### 2.3.2 Exposure variable

The exposure variable was the “perception of discrimination” by the head of the household (Yes/No). This variable was obtained from the ENPOVE 2022 question “Have you felt discriminated against since you arrived in Peru?”

#### 2.3.3 Covariates

The following variables were included as potential confounders of the association of interest, considering their availability in the ENPOVE 2022: Sex (female/male), age group (<30/30 to 39/40 to 49/50 to more years), wealth index (lower/middle/higher), educational level (higher/secondary/up to primary), worked the last week (yes/no), health insurance (yes/no), rented housing (yes/no), presence of children under five years old in the household (yes/no), presence of older adults in the household (yes/no), household size (one person/two to five/ six or more persons), physical or psychological limitation (yes/no), immigrant status (illegal/legal), city of residence (Lima and Callao/other city), Participation in community associations or meetings (yes/no), perception of unmet needs (yes/no). The wealth index variable was constructed from a principal component analysis that considered household characteristics and trends, following a previously reported methodology (Hernández-Vásquez et al., 2023). The variable “physical or psychological limitation” was constructed based on responses to the question: “Do you have a permanent limitation in any of the following areas?” Participants could report limitations in mobility (walking or using arms or legs), vision (even with glasses), speech or communication (even using sign language or other methods), hearing (even with hearing aids), cognitive abilities (understanding, concentrating, or remembering), and social interactions (due to thoughts, emotions, feelings, or behaviors). The variable was coded as “yes” if the respondent reported at least one limitation in any of these areas and “no” if no limitations were indicated. The variable “perceptions of unmet needs” was constructed based on responses to the question: “What are the three main needs that are not being met in your household today?” Participants could select up to three options from a predefined list, including food, access to healthcare, assistance in regularizing migration or refugee status, income generation or employment, education, non-food items (such as clothing or personal care products), housing, access to water and sanitation services, family reunification, recreation and leisure, or other specified needs. To create the binary variable, responses were coded as “yes” if at least one unmet need was reported and “no” if none of the listed needs were selected.

### 2.4 Statistical analysis

**Fig. 2:**
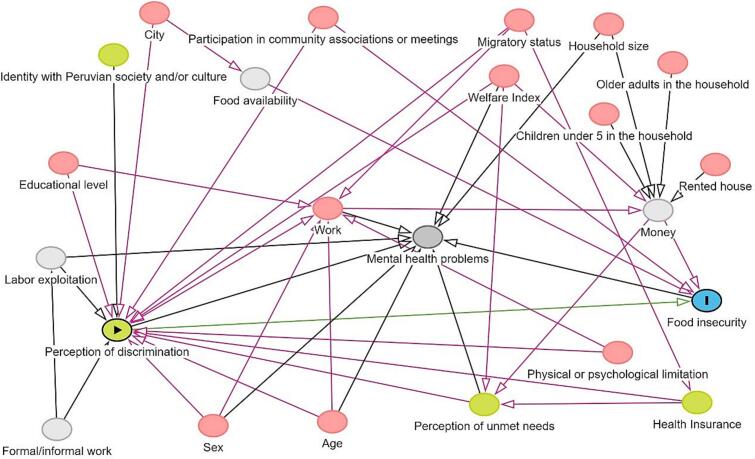
Directed Acyclic Graph of the association between perceived discrimination and household food insecurity among Venezuelan immigrants in Perú, 2022.

In the first instance, a descriptive analysis of the variables of interest (dependent, independent and covariates) was carried out using absolute frequencies and weighted percentages with their respective 95 % confidence intervals (CI). Subsequently, the weighted absolute and relative frequency distribution of exposure and outcome were tabulated according to potential confounding covariates. Finally, an analysis was performed using generalized linear models of the Poisson family using logarithmic linkage to estimate the association between the perception of discrimination and household food insecurity by means of prevalence ratios in a crude form and adjusted for confounding variables with the 95 % CIs, respectively. The present study evaluated the association of interest following an epidemiological and causal inference criterion, applying a Directed Acyclic Graph (DAG)-based approach. To account for confounding, three multiple regression models were performed, each using different variable adjustment sets, as identified by the web-based software DAGitty3.0 (Textor et al., 2011), after constructing the DAG representing the assumed causal structure of the association of interest (See Fig. 2). The model one adjustment set (City of Residence, Immigrant Status, Age, Sex, Educational Level, Participation in community associations or meetings, Perception of unmet needs at home, Physical or psychological limitation, Wealth Index) was considered as the main analysis. To assess the robustness of the association estimator and precision, we conducted sensitivity analyses using two additional adjustment sets: model two (City of Residence, Immigrant Status, Participation in community associations or meetings, Perception of unmet needs at home, Employment, Wealth Index) and three (City, Participation in community associations or meetings, Perception of unmet needs at home, Health Insurance, Employment, wealth Index). These adjustments sets were selected based on the DAG's minimal sufficient adjustment sets, ensuring that different valid combinations of confounders were used to block all backdoor paths between the exposure and outcome while avoiding overadjustment (i.e., including mediators) or introducing collider bias.

All analyses were performed taking into account a significance level of 5 % and with the use of Stata software version 17.0 StataCorp, College Station, TX, USA. All estimations took into account the complex sampling design and the expansion factors of ENPOVE 2022, configured using the svy command.

## 3 Results

The sample was composed of 3491 migrant Venezuelan heads of household (Table 1). The majority were males (64.9 %), between 30 and 39 years of age (38.9 %), with higher education (47.9 %) and legal immigrant status (75.1 %). Most of the participants worked in the last week (86.6 %). Only 24.2 % had health insurance. 95.6 % lived in a rented house, with the most frequent places of residence being Lima and Callao (83.8 %). Less than half (30.1 %) had children under five years of age in the household, and a smaller proportion had older adults (7.3 %). Similarly, the household size was of two to five persons (74.6 %). When evaluating the wealth index, most were in the lower wealth range (34.6 %). The perception of unmet needs was 91.2 % and only 2 % had some physical or psychological limitation. Moderate to severe food insecurity was reported in 39 % of the participants' households, while 35.6 % reported perceiving discrimination.

**Table 1:** Characteristics of the heads of the households among Venezuelan migrants residing in Peru included in the ENPOVE 2022 study (*N* = 3491).

Characteristics	n	% (95 % CI)
Sex		
Female	1262	35.1 (32.9–37.3)
Male	2229	64.9 (62.7–67.1)

Age group		
Less than 30 years	1207	32.9 (30.8–35.1)
30 to 39 years	1300	38.9 (36.8–41.1)
40 to 49 years	621	17.5 (16.0–19.2)
50 to more	363	10.6 (9.4–11.9)

Wealth Index		
Lower	1301	34.6 (32.2–37.1)
Middle	1172	33.9 (31.8–36.2)
Higher	1018	31.4 (29.2–33.7)

Educational level		
Higher	1584	47.9 (45.6–50.2)
Secondary	1483	43.3 (40.9–45.8)
Up to primary	424	8.8 (7.5–10.1)

Worked the last week		
No	490	13.4 (11.9–14.9)
Yes	3001	86.6 (85.0–88.1)

Health Insurance		
No	2784	75.8 (73.7–77.8)
Yes	707	24.2 (22.2–26.2)

Rented house		
No	174	4.4 (3.6–5.5)
Yes	3317	95.6 (94.5–96.4)

Children under 5 in the household		
No	2369	69.9 (67.8–71.9)
Yes	1122	30.1 (28.0–32.2)

Older adults in the household		
No	3225	92.7 (91.6–93.8)
Yes	266	7.3 (6.2–8.4)

Household size		
A person	608	17.5 (15.9–19.3)
2 to 5 persons	2574	74.6 (72.6–76.6)
6 or more persons	309	7.8 (6.7–9.1)

Physical or psychological limitation		
No	3431	97.9 (97.2–98.5)
Yes	60	2.0 (1.5–2.8)

Immigrant status		
Ilegal	1127	24.9 (22.9–26.9)
Legal	2364	75.1 (73.1–77.1)

City of residence		
Lima and Callao	1922	83.8 (82.7–84.9)
Other city	1569	16.2 (15.1–17.3)

Participation in community associations or meetings		
No	3087	88.2 (86.6–89.5)
Yes	404	11.8 (10.5–13.4)

Perception of unmet needs		
No	315	8.8 (7.6–10.2)
Yes	3176	91.2 (89.8–92.4)

Perception of discrimination		
No	2223	64.4 (62.3–66.5)
Yes	1268	35.6 (33.5–37.7)

Moderate to severe food insecurity		
No	2056	60.9 (58.6–63.3)
Yes	1435	39.0 (36.7–41.4)

Estimates include the weights and ENPOVE 2022 sample specifications.

The majority of households with moderate to severe food insecurity had heads of household who were women (46.7 %), in the lowest wealth index category (52.6 %), with secondary education (43.9 %), an illegal Immigrant status (49.3 %), and perception of unmet need (41.8 %). A higher frequency of moderate to severe food insecurity was identified in households in which the head had experienced discrimination (46.4 %), compared to those in which the household head had not (34.9 %) (Table 2).

**Table 2:** Characteristics associated with moderate to severe household food insecurity in the last month among Venezuelan migrants heads of household residing in Peru included in the ENPOVE 2022 study (N = 3491).⁎

Characteristics	Moderate to severe household food insecurity		*p*-value*
	No (*n* = 2056)	Yes (*n* = 1435)	
	n (%)	n (%)	
Sex			<0.01
Female	640 (53.3)	622 (46.7)	
Male	1416 (65.1)	813 (34.9)	

Age group			0.98
18 to 29 years	719 (61.4)	488 (38.6)	
30 to 39 years	757 (61.1)	543 (38.9)	
40 to 49 years	363 (60.7)	258 (39.3)	
50 to more	217 (59.7)	146 (40.3)	

Wealth Index			<0.01
Lower	587 (47.4)	714 (52.6)	
Middle	704 (62.1)	468 (37.9)	
Higher	765 (74.7)	253 (25.3)	

Educational level			<0.01
Higher	1017 (66.1)	567 (33.9)	
Secondary	834 (56.1)	649 (43.9)	
Up to primary	205 (57.1)	219 (42.9)	

Immigrant status			<0.01
Ilegal	556 (50.7)	571 (49.3)	
Legal	1500 (64.4)	864 (35.7)	

City of residence			0.14
Lima and Callao	1186 (61.5)	736 (38.5)	
Other city	870 (58.3)	699 (41.7)	

Physical or psychological limitation			0.08
No	2026 (61.3)	1405 (38.7)	
Yes	30 (46.6)	30 (53.4)	

Participation in community associations or meetings			0.67
No	1825 (61.2)	1262 (38.9)	
Yes	231 (59.7)	173 (40.4)	

Perception of unmet needs			<0.01
No	279 (89.4)	36 (10.6)	
Yes	1777 (58.2)	1399 (41.8)	

Discrimination perception			<0.01
No	1402 (65.0)	821 (34.9)	
Yes	654 (53.6)	614 (46.4)	

Estimates include the weights and ENPOVE 2022 sample specifications.

⁎ *p*-values were obtained using the Rao-Scott chi-squared test for categorical variables.

Most of the heads of households who perceived discrimination were female (39.3 %), had a higher education (39.9 %), lived in cities other than Lima and Callao (39 %), and perceived unsatisfied needs (36.4 %) (Table 3).

**Table 3:** Characteristics associated with perceived discrimination among Venezuelan migrants heads of household residing in Peru included in the ENPOVE 2022 study (N = 3491).

Characteristics	Perception of discrimination		p-value⁎
	No (*n* = 2223)	Yes (*n* = 1268)	
	n (%)	n (%)	
Sex			0.01
Female	759 (60.7)	503 (39.3)	
Male	1464 (66.5)	765 (33.5)	

Age group			0.34
18 to 29 years	742 (62.1)	465 (37.9)	
30 to 39 years	842 (65.8)	458 (34.2)	
40 to 49 years	389 (63.8)	232 (36.2)	
50 to more	250 (67.6)	113 (32.5)	

Wealth Index			0.37
Lower	813 (62.9)	488 (37.0)	
Middle	767 (66.4)	405 (33.6)	
Higher	643 (63.9)	375 (36.2)	

Educational level			<0.01
Higher	934 (60.1)	650 (39.9)	
Secondary	990 (66.8)	493 (33.2)	
Up to primary	299 (76.3)	125 (23.7)	

Immigrant status			0.22
Ilegal	740 (66.6)	387 (33.4)	
Legal	1483 (63.7)	881 (36.3)	

City of residence			0.03
Lima and Callao	1248 (65.1)	674 (34.9)	
Other city	975 (60.9)	594 (39.0)	

Physical or psychological limitation			0.69
No	2188 (64.4)	1243 (35.6)	
Yes	35 (67.1)	25 (32.9)	

Participation in community associations or meetings			0.56
No	1982 (64.7)	1105 (35.3)	
Yes	241 (62.7)	163 (37.3)	

Perception of unmet needs			0.01
No	233 (72.9)	82 (27.1)	
Yes	1990 (63.6)	1186 (36.4)	

Moderate to severe food insecurity			<0.01
No	1402 (68.7)	654 (31.3)	
Yes	821 (57.7)	614 (42.3)	

Estimates include the weights and ENPOVE 2022 sample specifications.

⁎ *p*-values were obtained using the Rao-Scott chi-squared test for categorical variables

In the crude regression model we identified that, perceived discrimination was associated with a higher probability of moderate to severe food insecurity in the household among Venezuelan migrants heads of household residing in Peru (Prevalence ratio: 1.33, 95 % CI: 1.19 to 1.47). This finding was maintained in adjusted model one (Adjusted prevalence ratio: 1.29, 95 % CI: 1.17 to 1.42) (Table 4). Similarly, models two and three showed results consistent with this finding (See table S2 of the supplementary material).

**Table 4:** Association between perceived discrimination and household food insecurity during the last month among Venezuelan migrants heads of household residing in Peru included in the ENPOVE 2022 study (*N* = 3491).

Characteristics	Crude model		Adusted model	
	PR	95 % CI	PR	95 % CI
Perception of discrimination				
No	Ref.		Ref.	
Yes	1.33	1.19–1.47	1.29	1.17–1.42

Estimates include the weights and ENPOVE 2022 sample specifications.

PR: Prevalence ratio. 95 % CI: 95 % confidence interval

Adjusted for city of residence, immigrant status, age group, sex, educational level, participation in community associations or meetings, perception of unmet needs, physical or psychological limitation, wealth index.

## 4 Discussion

This study evaluated the association between perceived discrimination by the head of household and moderate to severe food insecurity in Venezuelan migrant and refugee households in Peru. Nearly 40 % of household heads in ENPOVE 2022 reported perceived discrimination, and 36.4 % of households experienced moderate to severe food insecurity according to the FIES. In addition, household heads reporting perceived discrimination presented a 29 % higher probability of living in food-insecure households compared to those who had not perceived discrimination. Coordinated efforts by the Peruvian government and international organizations are needed to address this issue and reduce food and socioeconomic inequality.

Nearly four out of ten heads of households manifested having perceived discrimination since arriving in Peru. A similar result was found in a 2017 Chilean survey, where 26.92 % of migrant household migrants heads reported discrimination mainly for being foreigner, with increased likelihood due to skin color (8.23 times) and physical appearance (2.82 times) compared to the local population (Oyarte et al., 2023). Likewise, the International Organization for Migration reported that between February and March 2019, at the border checkpoints of Tacna and Tumbes, in southern and northern Peru, respectively, 29.9 % of Venezuelan migrants entering through Tumbes also suffered discrimination because of their nationality in some city along the route (97.5 %). On the other hand, among those who left Peru through Tacna, 37 % suffered some type of discrimination, especially because of their nationality (94.2 %), and to a lesser extent because of their economic situation (3.6 %) or their sex (1.3 %) (OIM, 2019). The perception of discrimination may also be linked to laws enacted in Peru. The law for the hiring of foreign workers governed by Legislative Decree No. 689 and its regulation of Supreme Decree No. 014–92-TR. in Peru establishes that only 20 % of the total number of employees may be foreigners and, in turn, their remuneration is limited to 30 % of the payroll salary of the total number of employees (Government of Peru, 1991). This law hinders migrants access to formal employment and violates equal work rights. As migrant status is a key driver of discrimination, host communities should be informed about migrant rights and the health impacts of social exclusion.

Almost half of the Venezuelan migrant and refugee households in Peru experience moderate to severe food insecurity. In Trinidad and Tobago, severe food insecurity reached 61.9 % among Venezuelan migrants, with high rates of skipping meals (81.76 %) and running out of food (77.14 %) (Saint Ville et al., 2022). Likewise, the study by Militao et al. showed that migrant household heads in the city of Maputo, there was a high prevalence of severe (34.4 %) and moderate (28.1 %) food insecurity (Militao et al., 2023). Another US study showed that food insecurity in non-Hispanic black households was higher (19.1 %) than the national average (10.5 %). Specifically, severe food insecurity affects 7.6 % of non-Hispanic black households compared to 3.3 % of non-Hispanic white households (Coleman-Jensen et al., 2020). This common issue among migrants, may be linked to sociodemographic factors such as education, as food insecurity is more prevalent in those with a high school education to those with university education (Militao et al., 2023; Tarraf et al., 2018). Food cost is another issue, as even employed migrants often prioritize quantity over nutritional value due to low income (Yeoh et al., n.d.; Vahabi and Damba, 2013). Also, most live in rented housing, which further limits the budget available for food (Vahabi and Damba, 2013). On the other side, a higher number of household members increases food insecurity due to income-dependency imbalance (Mansour et al., 2021). The factors, coupled with low household income and high rates of informal employment, can exacerbate the problem of food insecurity in the migrant population (Pico et al., 2021).

The main finding of the present study was that the perception of discrimination by the head of household was associated with a higher probability of moderate to severe food insecurity in the household of Venezuelan migrants and refugees. A 2018 Philadelphia study demonstrated that child and household food insecurity was increased in households with one or more experiences of discrimination in obtaining housing, public assistance, and medical assistance Among caregivers who experienced housing discrimination, 14 % were in households with food insecurity compared to 6 % who did not experience discrimination (Evans et al., 2020). In addition, in 2018, food insecurity rates for both Hispanic and non-Hispanic black households were described in a study which found that insecurity rates in these households were at least double those of non-Hispanic white households. This rate was associated with an increase in the frequency of racial discrimination along the lifetime, with there being a 5 % probability of presenting very low food security, even after adjusting for socioeconomic and demographic factors (Burke et al., 2018; Odoms-Young, 2018). Discrimination limits access to educational and employment opportunities, accompanied by social and economic consequences that lead to food insecurity. It also mentions discriminatory backgrounds which citizens have experienced at some point and which have negative repercussions on their present, hindering employment and consequently contributing to food insecurity (Odoms-Young, 2018). The development of programs to eradicate inequality towards foreigners and promote empathy and local integration is necessary.

This study has limitations to consider when interpreting results. First, its cross-sectional design prevents establishing causality. However, it is among the first to address this issue, as no longitudinal studies exist. Information bias is possible since key variables rely on self-reports of migration status, discrimination, and food quality, which may be influenced by social desirability. Still, food insecurity was measured with the FIES, which has strong psychometric properties. Perceived discrimination remains subjective and prone to misclassification. Interviewers or participants may have introduced errors, but training minimized this risk. Despite probabilistic sampling, selection bias may exist, as undocumented Venezuelan migrants could have avoided interviews. Lastly, only eight cities with higher migrant flows were included, but the ENPOVE 2022 sample represents at least 82 % of Venezuelan migrants and refugees in Peru.

## 5 Conclusions

The perception of discrimination among Venezuelan migrant households in Peru is linked to higher food insecurity. This study provides new insights by evaluating the association between perceived discrimination and moderate to severe food insecurity among Venezuelan migrant and refugee households in Peru, a topic that has not been widely explored in Latin America. Urgent actions are needed to address this issue and reduce food and socioeconomic inequality. Proposed measures include fostering social support groups, providing psychological assistance, addressing legal and rights education, and offering guidance on reporting discrimination. Revising labor laws to reduce illegal employment, especially in microenterprises, is essential. Additionally, leveraging technology and social networks can amplify migrants' voices and promote the economic benefits of their integration. Free nutrition programs focused on affordable, nutritious eating habits are also recommended.

## CRediT authorship contribution statement

**Darlene Milagros Rocha-Rojas:** Writing – review & editing, Writing – original draft, Visualization, Project administration, Investigation, Conceptualization. **Paola Fernanda Velásquez-Huamán:** Writing – review & editing, Writing – original draft, Visualization, Project administration, Investigation, Conceptualization. **Akram Hernández-Vásquez:** Writing – review & editing, Validation, Supervision, Methodology, Formal analysis, Data curation, Conceptualization. **Diego Azañedo:** Writing – review & editing, Writing – original draft, Validation, Supervision, Project administration, Methodology, Investigation, Formal analysis, Data curation, Conceptualization.

## Funding

Self-funded.

## Declaration of competing interest

The authors declare that they have no competing financial conflicts of interest or personal relationships that could have influenced the content of this article.

## Data Availability

Data is freely available at: https://proyectos.inei.gob.pe/microdatos/

## Appendix A Supplementary data

Supplementary material Image 1
