# Supplementary material for: Perception of discrimination by the head of the household and household food insecurity in Venezuelan migrants in Peru: Cross-sectional analysis of a population-based survey
Source: Prev Med Rep. 2025 Mar 26;53:103050. doi: 10.1016/j.pmedr.2025.103050 (PMC11995785; doi:10.1016/j.pmedr.2025.103050)
Supplement: Supplementary file 1 — Supplementary material [file mmc1.docx]

| The questions assess respondents' experiences and behaviors related to food access, specifically challenges encountered due to limited resources in the past 30 days. Has there been a time when, due to lack of money or other resources, you: |
| --- |
| 1. Worried about not having enough food to eat? |
| 1. Were unable to eat healthy or nutritious foods? |
| 1. Ate a small variety of foods? |
| 1. Had to skip a meal? |
| 1. Ate less than you thought you should? |
| 1. Ran out of food in your home? |
| 1. Felt hungry but did not eat? |
| 1. Did not eat for an entire day? |

**Table S1: FIES Questionnaire**

|  |  |  |  |  |  |
| --- | --- | --- | --- | --- | --- |

Table S2. Sensitivity analysis on the association between perceived discrimination and household food insecurity during the last month among Venezuelan migrants heads of household residing in Peru included in the ENPOVE 2022 (N=3491).

| Characteristics | | Multiple regression* | |
| --- | --- | --- | --- |
|  |  | PR | 95% CI |
| Model 2 | |  |  |
| Perception of discrimination | |  |  |
|  | No | Ref. |  |
|  | Yes | 1.29 | 1.17 - 1.43 |
| Model 3 | |  |  |
| Perception of discrimination | |  |  |
|  | No | Ref. |  |
|  | Yes | 1.29 | 1.17 - 1.43 |
| *Model 2 adjusted for city of residence, inmigrant status, participation in community associations or meetings, perception of unmet needs, worked the last week, wealth index.  *Model 3 adjusted for city of residence, participation in community associations or meetings, perception of unmet needs, health insurance, worked the last week, wealth index. | | | |
| Estimates include the weights and ENPOVE 2022 sample specifications. | | | |
| PR: Prevalence ratio. 95% CI: 95% confidence interval | | | |
